# Effect of Chinese outward FDI on youth unemployment in sub-Saharan Africa

**DOI:** 10.1371/journal.pone.0305482

**Published:** 2024-07-17

**Authors:** Junqi Liu, Ellis Chukwumerije Nwagu, Rongbing Liu, Qi Wang, Gouranga Chandra Debnath, Roni Bhowmik

**Affiliations:** 1 School of Finance and Trade, Liaoning University, Liaoning, China; 2 School of Business & Economics, United International University, Dhaka, Bangladesh; 3 School of Business, Guangdong University of Foreign Studies, Guangzhou, China; 4 Lancashire School of Business and Enterprise, University of Central Lancashire, Preston, United Kingdom; 5 Department of Business Administration, Daffodil International University, Dhaka, Bangladesh; University of Madeira / NOVA Lincs, PORTUGAL

## Abstract

This paper investigates the effect of Chinese outward foreign direct investment (FDI) on youth unemployment in sub-Saharan Africa (SSA) by using a modified labour demand model to identify the investment sources that are helpful for reducing youth unemployment in the region. The model is applied to a panel of 42 countries for the period 2003–2021 using random-effect, and generalized method of moment (GMM) estimators for robustness check. Our results suggest that Chinese FDI has direct short-term reducing effect on youth unemployment in SSA. The direction of China’s capital investment to infrastructure development and other labour-intensive activities leads to immediate reduction in youth unemployment. However, overtime, due to absence of linkages with domestic firms, and thus lack of demand effects, Chinese FDI becomes insignificant for employment creation. Our results also indicate that Other FDI does not lead to significant reduction in youth unemployment both currently and overtime. Our analysis gives partial support to the argument that the impact of Chinese FDI may differ from those of developed countries. Finally, we could not find evidence that the effect of FDI on employment depends on host country human capital and institutional quality. Several specifications of the empirical model are tested, and explanations are provided for the results. Policy implications are highlighted, especially the need to attract more job absorbing FDI and improve domestic absorptive capacity.

## 1. Introduction

An important part of international investment is the ongoing rise in outward foreign direct investment (FDI) from developing economies. The share of developing economies in global outward FDI flows has increased from 4.7% at the beginning of the new century to 30.8% by the end of 2022, after reaching 52.2% in 2022 [1]. China has been a major driver of this growth, contributing on average 36% of the annual FDI outflows from developing economies between 2017 and 2022 (See UNCTAD, 2023, Annex Table 1, pp 196–199) [1]. Its outward FDI now ranks among the top-2 in the world (MOFCOM, 2023) [1].

The emergence of Chinese outward FDI in many developing countries provides the hosts with additional sources of finance, technology and management resources for investment, formation of new firms, and potential creation of new jobs. Researchers have studied the determinants of outward FDI from developing and emerging countries, including China. Literature shows that Chinese outward FDI targets different range of countries, especially those in developing and emerging markets. These investments have various motivations, including expanding market presence, securing access to natural resources, acquiring strategic assets, and enhancing geopolitical influence [2–4]. However, the understanding of Chinese FDI’s impacts on host developing economies is limited. This is due to several structural and institutional characteristics of Chinese multinational enterprises (MNEs) that make them unique. While some aspects of Chinese MNEs align with other MNEs globally, Chinese MNEs stand out in two ways when conducting FDI in developing countries: (1) the significant role played by home country governments as institutional forces [2, 5]. For instance, the “go global” policy system sponsored by Chinese government provides several incentives to encourage domestic Chinese enterprises to conduct overseas investment, and sometimes the government gives financial support to selected enterprises to invest in specific countries and industries. (2) like other emerging market MNEs, the challenge of going abroad without superior technological and managerial resources compared to MNEs from advanced industrial countries [6]. These two differences can have implications on how Chinese outward investors affect the host developing countries. For instance, the technologies brought by Chinese investors may be more suited to the technology level of host developing countries. This can result in faster technology transfer.

On the other hand, Chinese investors, being relatively new to the game, may have limited experience in creating linkages with domestic enterprises. This may inhibit technology transfer. In addition, having strong home institutional backing combined with incentives and financial support, Chinese investors can provide more capital investments to host countries by investing in critical areas overlooked by traditional investors. Considering these structural and institutional differences, the rise in China’s outward FDI prompts the following questions: How does Chinese outward FDI affect host developing economies? How does the impact of Chinese outward FDI differ from the impact of other FDI?

This paper addresses this issue with a focus on the impact of Chinese outward FDI on youth unemployment in sub-Saharan Africa (SSA). The case of Chinese outward FDI to SSA is considered for several reasons. Firstly, China is a leading investor economy in the global scene and in Africa, it provides more greenfield projects to Africa, and created more jobs per project compared to other investor economies in Africa [7].

Secondly, youth unemployment is a critical issue in SSA. Over 60% of SSA’s population is younger than 25 years. Youth unemployment in the region has increased rapidly and job-creation programs initiated for the young people have had negligible impact. The recent COVID-19 pandemic has worsened the situation, reversing the little progress made during 2007–2018 period when many SSA countries registered high economic growth rates. Today, about one-fourth of SSA youths between the age 15–24 are unemployed [8], and this varies across countries, with countries such as Sierra Leone and South Africa having youth unemployment rates of 70% and 63% respectively [9]. While some of these unemployed young people are without education and training, many of them, especially those in lower-middle-income countries are well-educated and highly skilled. They remain unemployed due to the absence of suitable job opportunities that can utilize their skills. Creating new firms and new jobs becomes a necessity. It is therefore important to examine the different potential sources of new firms and job creation. Thirdly, empirical literature on the impact of Chinese outward FDI in Africa is limited. Although there is a growing body of literature on Chinese outward FDI in Africa since the 2010s, most of them focus on the purpose of Chinese FDI in Africa [10–14]. The few studies that analyse the economic impact focus on growth rates [15, 16], and those that explore employment effect are mainly qualitative studies [17, 18]. Thus, there is an empirical research gap on the employment effects of Chinese outward FDI in Africa.

This paper fills this empirical gap by using the most recent cross-country panel data for the 2003–2021 period. Although cross-country analyses have their limitations, the result of this study can indicate a better overall (average) understanding of Chinese outward FDI’s employment effect in Africa and increase our understanding of the impacts of outward FDI from developing and emerging market economies. Apart from using the most recent data and a large panel sample of 42 countries, this study has several distinguishing characteristics. First, it analyses the direct and indirect youth employment impact of Chinese outward FDI to SSA. Second, it utilizes a relatively clear empirical specification. It starts by building a theoretical model of labour demand based on the Cobb-Douglas production function to show the relationship between inward FDI and employment. Third, it estimates, as in previous studies, other factors that influence unemployment in SSA. Fourth, it performs separate regression models for Chinese FDI and other FDI, in order to compare and contrast the two models. Fifth, the study tests the effect on youth unemployment, which has been one of the most critical socio-economic challenge facing SSA in recent years. By using youth unemployment as the dependent variable, the finding will provide insight into a critical issue and will serve as a reference for African policymakers when designing policies targeting sustainable employment for the huge young population of SSA. Finally, the study performs robustness tests to check if the specifications are sensitive to the estimation model used. The main results indicate that Chinese FDI has a negative and significant direct effect on youth unemployment in SSA, while Other-FDI does not have a significant impact. Lagged values of Chinese FDI and Other FDI do not have significant impact on youth unemployment. Additionally, Domestic absorptive capacity of human capital and institutional quality have not enhanced FDI’s impact on youth unemployment rates. Moreover, Chinese FDI differ from Other FDI in the direct short-term effects only.

The rest of the paper is organized as follows. Section 2 reviews the literature, Section 3 describes model and methodology, Section 4 describes data and variables, Section 5 presents the results, and section 6 concludes the paper.

## 2. Literature review

There is a relationship between investment and employment. Traditional Keynesian theory suggests that increased investment activity, induced by positive demand shocks (resulting from expansionary fiscal and monetary policies), should lead to an increase in the level of employment [19]. Although Keynesian employment theory does not specifically address the role of FDI, it creates the expectation that FDI should affect employment, as FDI involves not only flows of investment funds but also long-term commitment to a productive activity in the host country. In this connection, researchers have studied the relationship between FDI and employment. However, there is still significant divergence in opinion among economists as regards the employment effects of FDI. The issue is there are several factors that influence the effects of FDI on employment [20], and there are various diverse channels through which these effects are transmitted [21].

To begin, FDI can boost employment growth directly when foreign subsidiaries and affiliates of MNEs create new jobs in their productive facilities. However, this depends on whether FDI involves greenfield investment or simply mergers and acquisitions [22]. Realized greenfield FDI is expected to have the greatest potential to directly generate new employment demand because it creates extra productive capacity by setting up new productive facilities. By comparison, the short-run effects of M&As may be insignificant, as it does not generate new employment demand [23], at most it can preserve employment by acquiring and restructuring ailing firms [24]. Direct employment generation also depend upon the ratio of labor to capital used by FDI firms in production. More jobs are expected to be created where foreign firms engage in labour-intensive activities [25], especially when the size of such FDI is large, the host country has abundant low-cost labour and a policy of attracting export-oriented FDI [20]. However, foreign entry in labour-intensive sectors through privatization can lead to job losses in the short run when the privatized firm restructures or lays off its excess labour to increase efficiency [26]. Direct employment creation effect of FDI may also be altered by the extent to which FDI substitutes for domestic investment [22]. Job losses are expected in the short-term when FDI replaces planned domestic investment. This occurs when domestic investors withdraw or reduce planned investment because they cannot compete with foreign investors [23].

Following from that, FDI can decrease employment through higher productivity due to higher productive capacity per unit of labour. MNEs are believed to possess ownership advantages, such as new technologies, expertise and management know-how, which enables them to generate higher productivity. Insofar as these ownership advantages are transferred to their foreign subsidiaries, the subsidiaries will gain higher productivity per worker, making their production process less labour intensive [27–29]. In this regard, foreign firms with higher labour productivity may create less employment than domestic firms.

Finally, inward FDI can affect employment indirectly by influencing domestic firms’ demand for employment through competition, productivity spillovers, and demand in upstream markets. If the presence of FDI leads to increased demand for locally produced inputs, domestic firms in supply sectors will increase output so as to service the increased demand. This would lead to employment growth [30]. However, through their interactions with foreign firms, domestic firms in supply markets may benefit from productivity spillovers which can make their production process less labour-intensive. This would reduce their demand for employment [21]. If the presence of foreign firms generates a competition pressure that forces domestic firms in the same sector to exit, employment demand in the domestic competitors may be reduced [25]. Finally, if domestic competitors buy inputs from local suppliers that enjoy linkages with foreign firms, or recruit local employees who received training and experience from foreign firms, domestic firms may benefit from knowledge spillovers and availability of high quality and cheaper inputs. These might lead to increase in their output and thus employment demand [31, 32].

As the different influencing factors and diverse transmission channels result in opposite effects, the net employment effect of FDI depends on the net creation of new capacity, productivity enhancements, and knowledge spillovers. Researchers argue that FDI can promote long-term growth in developing countries through knowledge spillovers and human capital development [33, 34]. De Mello adds that the growth enhancing effect of FDI depends on the extent to which FDI complements or substitutes domestic investment, while Borensztein et al. argues that realizing the growth benefits of FDI depends on the stock of human capital. Following from that, Pigato (2000) point out that for most African countries, the FDI that enhances domestic investment are those that provide human capital development, backward linkages, and physical, scientific and institutional infrastructure [35]. In this regard, for SSA countries, employment effect of FDI may depend mainly on net additions to physical capital and knowledge spillovers.

Empirical studies on the effect of FDI on employment provide conflicting results. Panel studies have been conducted for cross section of countries in different regions. Among the panel data studies, Vacaflores et al. (2017) examine the effect of FDI on employment in host countries using a global dataset comprising 897 MNEs and 5,641 foreign subsidiaries during 2006–2008 period [36]. Their findings reveal that FDI exerts positive and strong direct effect on employment in foreign affiliates that: (1) are not subjected to high levels of FDI disinvestment, (2) operate in host countries that have low FDI to GDP ratio, and (3) operate in manufacturing and mining sectors. This positive direct effect decreases as the affiliate firm grows. Further, their results suggest that the positive direct effect of FDI on employment creation based on the size of FDI, and differences in foreign subsidiaries, home and host countries. FDI between developing countries generates employment in host countries within foreign affiliates. FDI from developed to developing countries and vice versa also has positive direct effect on employment in the foreign affiliates. This finding reflects the heterogenous effects of FDI on employment.

In Asia, Farooq et al. (2024) applied ARDL regressions on 30 years of annual data from South Asian economies over the period 1990 to 2019 [37]. They reported that FDI inflow has strong positive impact on employment rate in south Asian region. In contrast, Mehmood et al. (2018) found that FDI has a significant and negative long-run effect on employment in South Asian countries, due mainly to FDI’s enhancement of growth through the channel of increased productivity, which lead to jobless growth [38]. Their study employed Fully Modified OLS techniques on a panel of south Asian economies from 1980–2017. However, Rizvi and Nishat (2009) examined the impact of FDI on employment in Pakistan, India and China [39]. They found that FDI does not have a significant effect on employment in the 3 Asian countries. They argued that although FDI filled the investment gaps and probably created jobs, but this was offset by fall in labour demand due to increased productivity and rationalization by foreign firms, especially in M&As FDI.

In Central and Eastern Europe, Jude and Silaghi (2016) applied fixed effects and GMM estimators on a sample of 20 CEEC countries during 1995–2012 for the purpose of investigating the impact of FDI on aggregate employment [26]. Their results suggest that FDI has a negative short-run effect and a positive long-run effect on employment. They argued that competition pressure and labour cost saving strategies lead to a negative short run effect on employment. However, the effect becomes positive in the long run, as foreign subsidiaries progressively create linkages with domestic firms and increase the local content of their production. Additionally, they argued that human capital played a critical role in enhancing the employment impact of FDI. The impact on employment is also affected by the type of FDI. Marinescu (2020) finds in the case of CEEC that greenfield FDI has very significant effect on job creation, while the effect of M&As FDI is uncertain, due to loss of jobs arising from efficiency measures [40].

In Latin America, Vacaflores et al. (2011) examines the role of FDI in improving employment rates during 1980–2006 [41]. Their findings reveal that FDI has a positive and significant effect on employment creation in host countries, driven mainly by its effect on male labour force. Additionally, the positive effect is very significant for less developed countries, times of low inflation, and for the long run. However, only countries with high levels of informality and below average FDI inflows gain from this effect.

In the case of Africa, Wall et al. (2018) explored the impact of three aspect of FDI, greenfield FDI, FDI flows and FDI stock, on total employment in Africa [42]. Their results reveal that both FDI inflow and FDI stock have very significant positive impact on total employment, while greenfield FDI has insignificant but positive impact on employment. They argue that the reason greenfield FDI does not have a significant impact on employment is because a large share of greenfield FDI to Africa is concentrated in extractive activities, which does not generally lead to significant employment creation and technology transfer. However, results from their sectoral analysis show that greenfield FDI, FDI inflow and FDI stock all have significant and positive effect on manufacturing employment, while for agricultural employment the impact is not significant but positive. Additionally, they show that human capital absorptive capacity is weak, while that of institutional quality is strong in enhancing FDI’s impact on employment. Their findings also point to the importance of developing domestic absorptive capacity in order to reap the benefits from FDI. While they focused on the impact of different aspects of FDI on total employment and sectoral employment, the source of FDI is equally important for employment in Africa [42]. Coniglio et al. (2015) examined the effect of FDI on employment and wages in SSA, using micro-level dataset covering 19 countries [43]. Their findings suggest that FDI creates more employment than domestic investment, but majority of the employment created are not skill-intensive. They also find that nationality of foreign firms matters for job creation in host countries, and there are differences between developed and developing country FDI in terms of the skill-intensity of their labour demand. Particularly, MNEs from global south created less skilled jobs, while those from global North created more high paying jobs. Additionally, they suggest that Chinese firms generate more jobs but pay lower wages for both skilled and less-skilled jobs compared to both domestic firms and other foreign firms.

Mixed evidence has been found in different subregions of SSA as well. Woldetensaye et al. (2022) explored the relationship between FDI and unemployment in five East African IGAD countries using data for the 1996–2021 period [44]. Applying random effect estimator, they found significant negative relationship between FDI and unemployment rate, suggesting that inward FDI promotes employment in the region. In contrast, Mkombe et al. (2021) found that the effect of FDI on youth unemployment is not significant in the case of southern Africa SADC region [45]. Their study applied FDGLS-Parks and PCSE estimators on a dataset covering 4 SADC countries during 1994–2017. They argued that the insignificant effect of FDI on youth unemployment was influenced by the mode of entry of FDI in the region such as M&As which causes less job creation compared to greenfield FDI. Finally, Aderemi et al. (2022) explored the long-run and short-run impact of FDI inflows on employment generation in Economic Community of West African States (ECOWAS) countries between 1990 and 2019 [46]. Their results indicate positive long-run relationship between FDI and employment in the region, driven mainly by capacity creation and linkage effects.

Studies of employment impact of Chinese FDI in Africa also produce mixed results. Boakye-Gyasi and Li (2015) estimated the impact of Chinese FDI on job creation in Ghana’s building and construction sector during 2000 to 2012 [47]. They find that Chinese FDI has significant direct positive effect on employment growth, mainly through the capital investment and capacity creation in area of infrastructure. Supporting the above finding, researchers argued that not only did Chinese firms create new jobs and absorbed local employees, they also promoted gender balance in employment [48]. In an interview-based study involving relevant host government officials in 5 SSA countries, scholar found that Chinese firms are perceived to create jobs through their heavy investments in labour-intensive sectors [49]. Furthermore, Khodeir (2016) applied fixed-effect regression on a panel dataset of 38 countries during 2007–2012 period with the objective to find the effect of Chinese FDI on employment in Africa [50]. He found a significant positive relationship between Chinese FDI and employment in Africa as whole and in SSA as a region. He highlighted the crucial role of education in the relationship, which increased employment by 5%.

In contrast, some studies have associated Chinese FDI in Africa with decreases in production and employment in certain domestic industries. Researchers argued that influx of cheap Chinese products in some cases displaced locally manufactured goods, leading to factory closures and job losses in specific sectors in several countries [51]. Zhang (2022) points to debt trap and lack of job creation as major disadvantage of Chinese investment to Africa [52]. Some of the researchers conclude that Chinese investment in Africa neither increases job quantity nor improves job quality, due to importation of workers from home country and creation of low-skilled jobs for local employees [18, 53]. Scholars argues that although Chinese firms have localized 89% of their workforce, most are low-level positions [17]. This limits the opportunity for training, higher salaries, and knowledge spillover to local employees. On the contrary, Oya and Schaefer (2023) argued that Chinese FDI creates jobs, contributes to skills and training of local workers, and pays wages as much as other firms in the same sector [54]. With this conflicting evidence, the employment effect of Chinese FDI in Africa is still not confirmed.

### Hypothesis development

Following the Keynesian employment theory that a positive relationship exists between investment and employment through positive demand effects [19], and postulations in the literature that FDI can increase employment quantity directly by adding to net capital and indirectly through forward and backward linkages and economy-wide multiplier effects [55], we hypothesize as follows:

H1. Chinese FDI to SSA may reduce youth unemployment.

Potential benefits from FDI are not realized automatically. Attracting FDI is only the first step, host countries must be able to induce the transfer of, and exploit the bundle of resources brought by FDI, such as knowledge, skills, technology and access to global markets [20]. Researchers have argued in line with the absorptive capacity (AC) theory that host countries must develop certain domestic capabilities in order to reap the gains from FDI, such as knowledge and technology spillovers [56–59]. AC refers to the ability of host countries to identify, mediate, assimilate and take advantage of the potential benefits of FDI [60]. Empirical findings support the significant role of AC in acquiring the benefits of FDI. Moralles and Moreno (2020) use data of Brazilian firms to examine whether FDI productivity spillovers depend on local AC, they find that positive spillovers from FDI may be captured by firms with high AC [61]. Sultana & Turkina (2020) test whether the AC of host countries matter in the relationship between FDI and technological advancement, they find that domestic AC plays a strong moderating role [59]. Chen et al. (2011) finds that Chinese industries with high AC were equipped to exploit the spillovers from FDI [62]. Marcin (2008) reports that the size of spillovers to local firms depends on their AC [56]. Girma (2005) shows that the gains from FDI increases with domestic AC until a threshold level is reached [60]. The literature shows several elements that serve as important AC in interacting with FDI to enhance FDI’s beneficial impact on host economies. These may include human capital, financial development, infrastructure quality, trade openness, institutional quality, FDI policy, innovation and knowledge intensity. Our interest is in human capital and institutional quality.

Human capital can not only boost creativity, labour efficiency and productivity of domestic enterprises through technological advancement, but can also enable domestic enterprises to learn from foreign-owned firms and to acquire the benefits from FDI. Borensztein et al. (1998) investigates the effects of FDI on growth for a cross-section of host developing countries and finds that FDI promotes growth through the channels of capital accumulation and higher productivity growth, which are dependent on the stock of human capital [33]. Moreover, Jude and Silaghi (2016) find that human capital plays a critical role in enhancing FDI impact on employment in the case of CEEC countries [26].

H2. The effect of Chinese FDI on youth unemployment in SSA depends on the host country’s stock of human capital

Institutional quality also serves as critical AC that is needed to capture the benefits of FDI. To begin with, good institutions such as existence of rule of law, political stability, corruption control, property rights, enforcement of contracts, etc., encourages investment [63]. It can lower transaction costs and create economic environment that promotes growth and attracts FDI [64]. Furthermore, institutional quality can strengthen the ability of host countries to absorb the resources brought by FDI [65, 66]. Jude & Levieuge (2017) argue that institutional quality modulates capital accumulation and knowledge spillovers, which are the two main channels of FDI impact on economic growth [67]. Adegboye and Okorie (2023) find that weak institutions hinder host countries from reaping the benefits of FDI while strong institutions enhance it [68]. Cassimon et al. (2022) report that quality institutions play strong mediating role in the relationship between foreign capital inflows (including FDI) and selected social variables in host countries [69]. Thus, following the AC argument, we hypothesize as follows:

H3. The effect of Chinese FDI on youth unemployment in SSA depends on the host country’s institutional quality.

FDI from different countries may have varying effects on host economies. FDI may be classified as expansionary and defensive types [70, 71]. Chen and Ku (2000) argue that expansionary FDI enters the host country to exploit the investing firm’s ownership advantages, such as new technology and know-how, whereas, defensive FDI seeks cheap labour in the host country with the goal of reducing the cost of production [72]. They further suggest that FDI from developed countries have the features of expansionary FDI, while those from developing and emerging countries are more of the defensive type. In addition, expansionary FDI tends to target capital intensive industries [73], and they can contribute greater knowledge and technology transfers to host countries [74, 75]. By contrast, researchers suggest that defensive FDI tend to target labour-intensive activities in line with the global factory theory [76, 77]. In this regard, FDI from developed countries, tending to have high capital and technology-intensity, may create higher-skilled (may be fewer in number) jobs compared to those from developing and emerging countries. In contrast, FDI from developing and emerging countries, being more labour-intensive, may generate greater job quantity than those from developed countries. Moreover, in the specific case of China, several analyses of its outward FDI using the institutional and structural aspects of the theory of push factors (which identifies the driving forces of FDI from the home country perspective), show that Chinese outward FDI has some special characteristics. For example, Peng (2012) finds that the governments play significant role as an institutional force, and that there is absence of significantly superior technological and managerial resources compared to developed country MNEs [78]. The differences in outward FDI objective, characteristics and propelling forces shapes the expectation that FDI from different countries may have varying effects on host economies. Based on the foregoing theories, we hypothesize as follows:

H4. The employment effects of Chinese FDI to SSA may differ from that of other FDI.

## 3. Model and methodology

This article contains no studies with human participants or animals performed by any of the authors. To model the impact of Chinese outward FDI on youth unemployment in SSA, we adopt the theoretical framework used in Mkombe et al. (2021) [45]. The model is derived from a labor demand function according to the Cobb-Douglas production function as shown below–

$$Y_{it}=A^{\psi }K_{it}^{\eta }L_{it}^{\beta }$$ (1)

Where Y is real output, K is the stock of capital, L is the quantity of labor (employment), i represents country and t is time, A is the stock of technology, η and β are the output elasticities (and output shares) of capital and labor, respectively. The coefficient ψ enables *K* and *L* to affect the efficiency of *A* in the production process. Profit maximization model suggests selecting the optimal capital so that the cost of capital (C) equals the marginal revenue product of capital and the wage (W) equals the marginal revenue product of labor. Due to difficulty in estimating national capital stock, we remove capital stock from Eq (1) following Jude and Silaghi (2016) [26]. Removing the capital stock from Eq 1 leads to -

$$Y_{it}=A^{\psi }{\left(\frac{\eta }{\beta }E_{it}\frac{W_{it}}{C_{it}}\right)}^{\eta }L_{it}^{\beta }$$ (2)

*E* represents the employment level. Eq (2) is transformed to get the labor demand function by taking logarithms on the left and right sides of the equation and rearranging the terms as follows:

$$lnL_{it}=\emptyset_{0}+\emptyset_{1}{\ln Y}_{it}+\emptyset_{2}ln\frac{W_{i}}{C_{i}}$$ (3)

Where $\emptyset_{0}=-\left(\lambda ln\;A+\alpha +ln\;\alpha -\alpha \;ln\;\beta \right)/(\alpha +\beta ),\;\emptyset_{1}=1/\left(\alpha +\beta \right)$ and $\emptyset_{2}=\alpha /(\alpha +\beta ).$

Inward foreign direct investment affects the technical efficiency parameter and stimulates sustained increases in the technical efficiency of production through technology diffusion [26, 33]. Thus, considering technological change stimulated by inward foreign direct investment, technical efficiency can be modelled as a function of foreign direct investment as follows:

$$A_{it}=e^{\phi_{0}T_{i}}{IFDI}_{it}^{\phi_{1}}$$ (4)

Where IFDI is the stock of inward foreign direct investment in country *i* at time *t*, T is the time trend and $\phi_{0}\;\phi_{1}>0$. The relationship between labor (employment) and inward foreign direct investment is determined by taking the logarithm of *A*_it_ and substituting in the labor demand function in Eq (3) as follows;

$$lnL_{it}=\zeta +\emptyset_{1}lnY_{it}+\emptyset_{2}ln\frac{W_{i}}{C_{i}}+\emptyset_{3}ln{FDI}_{it}+\emptyset_{4}T$$ (5)

Where $\zeta =-\left(\alpha \;ln\;\alpha -\alpha \;ln\;\beta \right)/(\alpha +\beta );\;\emptyset_{3}=\mu \phi_{1};\;\emptyset_{4}=\mu \phi_{0};\;\mu =-\lambda /(\alpha +\beta ).$

Therefore, regarding unemployment as the opposite of employment, Eq (5) can indicate the relationship between inward FDI and unemployment if the right-hand side of Eq (5) is represented by the opposite signs.

To study the impact of China’s outward FDI on youth unemployment in SSA, the study employed panel data estimation techniques, and followed researcher work [26, 45, 79], in specifying the empirical model. However, since we are interested in the effect of China’s outward FDI to SSA, the FDI variable is separated into two, namely FDI from China and FDI from others. Furthermore, we include several control variables in order to capture the different factors that influence unemployment rate in SSA. The empirical model is specified as follows:

$${YU}_{it}=\beta_{0}+\beta_{1}{FDICHINA}_{it}+\beta_{2}{FDIOTHERS}_{it}+\beta_{3}{GDP}_{it}+\beta_{4}{INV}_{it}+\beta_{5}{POP}_{it}+\beta_{6}{HC}_{it}+\beta_{7}{INFL}_{it}+\beta_{8}{INFR}_{it}+\beta_{9}{OPEN}_{it}+\beta_{10}{AGRVA}_{it}+\beta_{11}{INST}_{it}+\beta_{12}{GAPCHINA}_{it}+\beta_{13}{GAPUS}_{it}+\beta_{14}{TNRR}_{it}+\nu_{it}$$ (6)

Where β_0_ is the intercept, β_1_ to β_15_ are the coefficients of *FDICHINA*, *FDIOTHERS*, *GDP*, *INV*, *POP*, *HC*, *INFL*, *INFR*, *OPEN*, *AGRVA*, *INST*, *GAPCHINA*, *GAPUS* and *TNRR*, respectively. YU is youth unemployment, FDICHINA is Chinese FDI to SSA, FDIOTHERS is FDI from the world (excluding China) to SSA, GDP is GDP growth, INV is domestic investment, POP is population growth rate, HC is human capital, INFL is the rate of inflation, INFR is infrastructure, OPEN is trade openness, AGRVA is agricultural value added, INST is institutions, GAPCHINA is technology gap from China, GAPUS is technology gap from the leader country (United States), TNRR is economic rents from natural resources, ν_it_ (μ_it_ + ε_it_) is the error term that includes errors in the youth unemployment measure μ_it_ (the combined time series and cross section error component) + ε_it_ (the cross section or individual specific component), *i* is individual countries in SSA region and t is the year from 2003 to 2021. Furthermore, following the absorptive capacity (AC) argument, we include interactive variables on the right-hand side of the equation to test the moderating role of human capital and institutional quality in the effect of FDI on youth unemployment. The addition gives the following equation:

$${YU}_{it}=\beta_{0}+\beta_{1}{FDICHINA}_{it}+\beta_{2}{FDIOTHERS}_{it}+\beta_{3}{GDP}_{it}+\beta_{4}{INV}_{it}+\beta_{5}{POP}_{it}+\beta_{6}{HC}_{it}+\beta_{7}{INFL}_{it}+\beta_{8}{INFR}_{it}+\beta_{9}{OPEN}_{it}+\beta_{10}{AGRVA}_{it}+\beta_{11}{INST}_{it}+\beta_{12}{GAPCHINA}_{it}+\beta_{13}{GAPUS}_{it}+\beta_{14}{TNRR}_{it}+\beta_{15}{\left(HC\times FDI\right)}_{it}+\beta_{16}{\left(INST\times FDI\right)}_{it}+\nu_{it}$$ (7)

Where *(HC × FDI)* is the interaction between human capital and FDI and *(INST × FDI)* represent interaction between institutional quality and FDI.

## 4. Data and variables

### 4.1 Variables

Since youth unemployment, which occurs when a youth is without work but available for and seeking employment, is part of the overall unemployment in a country, we consider the factors influencing unemployment as factors affecting youth unemployment.

The FDI received from China and those from other countries are our main independent variables of interest. As discussed in previous sections, inward FDI is a source of capital, technology, management knowledge and other organizational resources, which can add to the production capacity of the recipient country and generate new employment opportunities directly and indirectly [23]. In the theoretical model developed by Agosin and Mayer (2000), inward FDI together with domestic investment, forms the total investment undertaken by countries [80]. Thus, FDI should have all the expected favorable effects of domestic investment plus more, as the providers of FDI are assumed to possess superior abilities and ownership advantages which can spillover to the domestic economy. This study therefore assumes that Chinese FDI and other FDI into SSA is negatively related to youth unemployment.

Economic growth (GDP growth) is one of the main determinants of unemployment. According to Okun’s law, an inverse relationship exists between unemployment and output growth [71]. So, a high rate of economic growth reduces unemployment. In SSA, the studies of those [45, 79] confirm that economic growth reduces unemployment rate. Therefore, this study expects a negative relationship between economic growth (proxied by GDP growth rate) and youth unemployment. Domestic investment is another factor that influences unemployment. Keynes (2018) suggests the existence of a positive relationship between investment and employment, through positive demand effects [19]. Through increased capital investments, new firms and economic activities are created which can demand labor and increase employment opportunities for the labor force. Researchers shows that domestic investment proxied by gross fixed capital formation reduces unemployment [45, 81, 82]. Thus, we assume that a high rate of domestic investment, proxied by gross fixed capital formation, has a negative effect on youth unemployment.

Another key determinant of unemployment is inflation. Based on the “Philips curve relationship” which found the existence of inverse relationship between wage growth and unemployment [83], macroeconomists have established that inflation and unemployment are negatively related [84, 85]. Thus, this study also assumes a negative relationship between youth unemployment and inflation.

Another key determinant of unemployment is population growth. Previous studies indicate a positive relationship between population and unemployment [79]. Thus, this study expects a positive relationship between youth unemployment and population growth. Human capital is another factor that affects unemployment. Strong human capital and fast economic growth can lead to better employment opportunities. Human capital is the stock of productive skills, talents, knowledge, expertise and health of a country’s labour-force, which is accumulated through education, training and good healthcare. Human capital increases creativity and labour efficiency, hence economists associate human capital with high-skills, high-earnings, high economic growth rate [86, 87], and employment growth and stability [88]. Moreover, Samiullah (2014) and Mimi et al. (2022) find that human capital reduces unemployment in host countries [89, 90]. This study therefore expects a negative relationship between unemployment and human capital. The study also considers technology gap, economic openness to international trade as independent variables. Since investors from developing countries are assumed to use technologies that are more appropriate for developing countries [91], we hypothesize that technology gap with China reduces unemployment, while technology gap with the leader country (US) increases unemployment. Economic openness has been linked to economic expansion since the time of classical economics such as Adam Smith and David Ricardo. Economic openness can contribute to poverty reduction and stimulate the private sector to create jobs through the channels of market size expansion and increased productivity and innovation by introducing local firms to world-class competition, skills and technology [92]. However, openness could also hamper development and lead to loss of jobs where the economy is not competitive enough. Therefore, this study assumes that economic openness can reduce or increase unemployment.

The study also includes several other factors that affect unemployment in SSA, such as institutional quality, human capital, infrastructure, agricultural productivity and natural resources exploitation. In many SSA countries, there is inadequate infrastructure, such as poor transportation networks, electricity supply, internet facilities, etc., which can constrain economic activities and private investment, leading to fewer or no employment opportunities [93]. Institutional quality is another variable that affects economic performance, and hence employment. Efficient institutions can not only improve the investment climate and attract more FDI [64] but can also promote economic growth [64, 71], employment and living standard [26, 69]. Therefore, this study expects institutional quality to have a negative effect on youth unemployment.

Furthermore, over half of FDI conducted by developed country MNEs into Africa target natural resources or the extractive industry [94]. Based on the global factory theory, the MNEs export the extracted resources to other locations for processing or for use as raw materials. This type of FDI generates only few direct jobs due to its capital intensity and use of foreign expatriate workers but may generate indirect jobs through linkages and infrastructure construction [95]. Thus, natural resource endowed countries attract resource seeking FDI, which may help develop the extractive industry and may generate employment opportunities. This study therefore assumes that natural resource availability reduces unemployment. Most SSA countries still practice subsistence agriculture. Some emerging markets MNEs, such as Chinese investors tend to target the Agric sector which are often neglected by developed country MNEs due to perceived risks. These emerging market investors, often backed by state financial support, take the risks, and bring some level of technology which helps increase productivity in the agriculture sector and may generate jobs directly and indirectly. This study therefore assumes that better agricultural productivity reduces unemployment.

Moreover, following the AC theory, the interaction of FDI with human capital and institutional quality can affect FDI’s impact on employment. Apart from their standalone effect on employment, human capital and institutional quality play a critical moderating role in FDI impact on economic growth which occur through capital accumulation and technology spillovers [33, 67]. Therefore, this study expects FDI’s interaction with human capital and institutional quality (the interactive variables) to have a negative effect on unemployment. Table 1 presents the variables proxy and definitions.

**Table 1:** Variables.

Variable	Definition	Expected effect	Data source
Youth unemployment	Unemployment, youth total (% of total labor force ages 15–24) (modeled ILO estimate)		World Bank https://data.worldbank.org/indicator/SL.UEM.1524.ZS
FDI China	Chinese outward FDI stock in SSA	(-)	CARI http://www.sais-cari.org/chinese-investment-in-africa
FDI other	Inward FDI stock less stock of FDI received from China	(-)	UNCTAD, Authors https://unctadstat.unctad.org/datacentre/dataviewer/US.FdiFlowsStock
GDP growth	GDP growth (annual %)	(-)	World Bank https://data.worldbank.org/indicator/NY.GDP.MKTP.KD.ZG
Domestic invest	Gross fixed capital formation (% of GDP)	(-)	World Bank. https://data.worldbank.org/indicator/NE.GDI.FTOT.ZS
Population growth	Population growth (annual %)	(+)	World Bank https://data.worldbank.org/indicator/SP.POP.GROW
Human capital	School enrollment, secondary (% gross)	(-)	World Bank. https://data.worldbank.org/indicator/SE.SEC.ENRR
Inflation	Inflation, GDP deflator (annual %)	(+/-)	World Bank. https://data.worldbank.org/indicator/NY.GDP.DEFL.KD.ZG
Infrastructure	Individuals using the Internet (% of population)	(-)	World Bank https://data.worldbank.org/indicator/IT.NET.USER.ZS
Openness	Trade (% of GDP)	(-/+)	World Bank https://data.worldbank.org/indicator/NE.TRD.GNFS.ZS?iframe=true
Agriculture	Agriculture, forestry, and fishing, value added (% of GDP)	(-)	World Bank https://data.worldbank.org/indicator/NV.AGR.TOTL.ZS
Institutional quality	Average estimate of the six WGI variables*	(-)	Authors based on the WGI
Technology Gap China	The gap between the Chinese GDP per capita and host country GDP per capita divided by host country GDP per capita	(-)	Authors based on World Bank data, https://data.worldbank.org/indicator/NY.GDP.PCAP.KD
Technology Gap USA	The gap between the US GDP per capita and host country GDP per capita divided by host country GDP per capita	(+)	Authors based on World Bank data, https://data.worldbank.org/indicator/NY.GDP.PCAP.KD
Natural resources	Total natural resources rents (% of GDP)	(-)	World Bank https://data.worldbank.org/indicator/NY.GDP.TOTL.RT.ZS
HC × FDI	Human capital variable times relevant FDI variable	(-)	Authors
INST × FDI	Institutional quality variable times relevant FDI variable	(-)	Authors
	*The six WGI variables		
Control of corruption	Control of Corruption: Estimate	(-)	WGI. https://data.worldbank.org/indicator/CC.EST
Government effectiveness	Government Effectiveness: Estimate	(-)	WGI. https://data.worldbank.org/indicator/GE.EST
Political stability	Political Stability and Absence of Violence/Terrorism: Estimate	(-)	WGI. https://data.worldbank.org/indicator/PV.EST
Regulatory quality	Regulatory Quality: Estimate	(-)	WGI. https://data.worldbank.org/indicator/RQ.EST
Rule of law	Rule of Law: Estimate	(-)	WGI. https://data.worldbank.org/indicator/RL.EST
Voice & accountability	Voice and Accountability: Estimate	(-)	WGI. https://data.worldbank.org/indicator/VA.EST

### 4.2 Data

The study selects forty-two SSA countries as the sample for this analysis. The selection of study sample is limited by data availability. Table 2 shows the list of countries in the study sample.

**Table 2:** List of countries in the study.

Angola	Cote d’Ivoire	Liberia	Senegal
Benin	Djibouti	Madagascar	Sierra Leone
Botswana	Equatorial Guinea	Malawi	South Africa
Burundi	Eritrea	Mali	Sudan
Cabo Verde	Ethiopia	Mauritania	Tanzania
Cameroon	Gabon	Mauritius	Togo
Central African Republic	Gambia	Mozambique	Uganda
Chad	Ghana	Namibia	Zambia
Comoros	Guinea	Niger	Zimbabwe
Congo, Dem. Rep.	Kenya	Nigeria	
Congo, Rep.	Lesotho	Rwanda	

Data of Chinese FDI to Africa is compiled annually by China Africa Research Initiative (CARI) based on the Statistical Bulletins of China’s Outward FDI published by China’s MOFCOM. The data is available for download in excel format at http://www.sais-cari.org/chinese-investment-in-africa. Data of FDI inward stock is obtained from UNCTAD data center, as shown in Table 2. Data of “governance” is computed by authors based on the six Worldwide Governance Indicators (WGI) estimates. The estimate for each WGI range from -2.5 to 2.5, with 2.5 indicating highest-quality governance. All other data are obtained from the World Bank’s WDI database.

#### 4.2.1 Descriptive statistics

Descriptive statistics summarize and organize dataset features. In empirical research, it is important to describe characteristics of the relevant dataset before testing and estimating them. This section presents the descriptive statistics of our dataset.

Unemployment rate has the average value of 15.04% in the 42 SSA countries with a minimum value of 0.47% in Niger in year 2011 and a maximum value of 78.78 in Djibouti in year 2020. Chinese outward FDI stock as a share of host country GDP in SSA averaged 1.98% with a minimum of 0.0002% in Republic of Congo in year 2003 and a maximum of 17.41 in Eritrea in year 2016. The stock of inward FDI (excluding those from China) as a share of GDP in SSA has the average of 46.39% with a minimum of -58.58 in Djibouti in 2020 and a maximum of 783.58% in Liberia in 2010. The summary statistics for all data are presented in Table 3.

**Table 3:** Descriptive statistics of the regression variables for SSA, 2003–2021.

Variable	Observations	Mean	Std. Dev.	Min	Max
Youth unemployment	798	15.04	14.41	0.47	78.78
FDI China	798	1.98	2.68	0.00	17.41
FDI others	798	46.39	83.61	-58.58	783.58
GDP growth	798	4.56	5.98	-36.39	59.53
Domestic investment	798	24.20	10.71	1.53	85.10
Population growth	798	2.52	0.89	-0.40	5.63
Human capital	472	44.76	22.34	7.16	114.72
Inflation	777	9.74	28.18	-21.17	604.95
Infrastructure	772	13.39	16.40	0.03	73.50
Openness	725	68.53	36.84	4.13	348.00
Agriculture	798	22.59	15.19	0.89	76.33
Governance	798	-0.67	0.60	-1.73	0.87
Technology gap China	798	6.50	6.07	-0.72	40.11
Technology gap USA	798	63.42	47.13	2.74	223.77
Natural resources	786	11.65	10.72	0.00	56.29
FDI China × HC	472	76.63	127.57	0.00	910.95
FDI Other × HC	472	2055.68	4856.76	-3215.79	42699.99
FDI China × Institutional quality	798	-1.48	3.10	-28.23	7.83
FDI Other × Institutional quality	798	-30.41	83.24	-1066.34	226.68
*Control of corruption*	798	-0.67	0.60	-1.65	1.25
*Govt effectiveness*	798	-0.80	0.59	-1.88	1.15
*Political stability*	798	-0.56	0.86	-2.70	1.20
*Regulatory quality*	798	-0.71	0.61	-2.28	1.20
*Rule of law*	798	-0.71	0.61	-1.87	1.02
*Voice & accountability*	798	-0.59	0.75	-2.23	0.98

#### 4.2.2 Panel unit root tests

When conducting panel regression analysis, it is important to perform tests for unit roots (or (stationarity) in the panel datasets, to check that the dataset meets the regression assumptions and to prevent spurious results. Five different unit root tests are performed, namely Levin-Lin-Chu (LLC) test, Breitung test, Im-Pesaran-Shin (IPS) test, Fisher-type test based on Philips-Perron (PP) test, and Fisher-type test based on augmented Dickey-Fuller (ADF) test. LLC and Breitung unit root tests assume a common autoregressive parameter (or common unit root process). Thus, to perform the LLC and Breitung tests the panels must be balanced. IPS, PP, and ADF unit root tests assume panel-specific autoregressive parameters (or individual unit root processes), thus they can be applied to unbalanced panels. All five tests have as the null hypothesis that all the panels contain a unit root. LLC and Breitung have as the alternative hypothesis that panels are stationary, while IPS, PP, and ADF unit root tests have as the alternative hypothesis that at least one panel is stationary. Table 4 presents the unit root test results. I (0) stands for stationarity at level or order zero, and I (1) indicates stationarity at first difference or order one.

**Table 4:** Panel unit root tests for Chinese OFDI and youth unemployment in SSA.

	Tests assuming common unit root process		Tests assuming individual unit root process		
Variables	LLC	Breitung	IPS	PP (Fisher)	ADF (Fisher)
	T-statistic	T-statistic	W-statistic	Chi^2^ statistic	Chi^2^ statistic
Youth unemployment	-6.125*** I(1)	-6.588*** I(1)	-7.783*** I(1)	48.737*** I(1)	18.270*** I(1)
FDI from China	-1.686** I(0)	-11.584*** I(1)	-8.674*** I(1)	52.727*** I(1)	16.156*** I(1)
FDI from others	-10.172*** I(1)	-6.206*** I(1)	-10.263*** I(1)	1.654** I(0)	19.163*** I(1)
GDP growth	-6.163*** I(0)	-4.339*** I(0)	-6.621*** I(0)	31.575*** I(0)	11.307*** I(0)
Domestic Investment	-2.481*** I(0)	-3.161*** I(0)	-13.187*** I(0)	4.096*** I(0)	3.102*** I(0)
Population growth	-4.138*** I(0)	-3.480*** I(0)	-2.795*** I(0)	14.080*** I(1)	6.959*** I(0)
Human capital	N/a	N/a	N/a	3.616*** I(0)	N/a
Inflation	-7.504*** I(0)	-6.206*** I(0)	-7.764*** I(0)	23.652*** I(0)	16.204*** I(0)
Infrastructure	-6.329*** I(0)	-8.522*** I(0)	-4.430*** I(1)	14.546*** I(1)	5.177*** I(1)
Openness	N/a	N/a	-15.448*** I(0)	2.162** I(0)	5.635*** I(0)
Agriculture	-4.802*** I(0)	-6.615*** I(1)	-2.293** I(0)	5.123*** I(0)	4.108*** I(0)
Institutional quality	-5.033*** I(0)	-5.031*** I(1)	-2.109** I(0)	3.877*** I(0)	3.639*** I(0)
Technology-Gap China	-2.055** I(1)	-4.986*** I(1)	-3.604*** I(1)	23.403*** I(1)	2.243** I(0)
Technology-Gap USA	-6.955*** I(0)	-2.971*** I(0)	-1.667** I(0)	3.329*** I(0)	3.996*** I(0)
Natural resources	N/a	N/a	-2.744*** I(0)	3.602*** I(0)	2.543*** I(0)
FDI China × HC	N/a	N/a	N/a	23.89*** I(1)	N/a
Other FDI × HC	N/a	N/a	N/a	24.86*** I(1)	N/a
FDI China × Inst qty	-2.128** I(0)	-2.232** I(0)	-8.870*** I(1)	58.35*** I(1)	16.18*** I(1)
Other FDI × Inst qty	-1.753** I90)	-6.213*** I(0)	-9.646*** I(1)	2.276** I(0)	2.135*** I(0)
*Control of corruption*	-2.845*** I(0)	-2.289*** I(0)	-13.219*** I(0)	2.454*** I(0)	2.851*** I(0)
*Govt effectiveness*	-3.843*** I(0)	-4.083*** I(1)	-4.033*** I(0)	10.048*** I(0)	6.437*** I(0)
*Political stability*	-3.933*** I(0)	-1.6611** I(0)	-2.693*** I(0)	9.021*** I(0)	4.287*** I(0)
*Rule of law*	-5.019*** I(0)	-2.314** I(0)	-2.557*** I(0)	3.095*** I(0)	3.128*** I(0)
*Regulatory quality*	-2.955*** I(0)	-3.651*** I(0)	-14.518*** I(1)	1.837** I(0)	2.175** I(0)
*Voice & accountability*	-6.225*** I(0)	-1.718** I(0)	-11.559*** I(1)	3.848*** I(0)	11.206*** I(0)

Note

*** and ** represent significance at 1% and 5% respectively. N/a means the test statistic was not computed due to unbalanced panel or normality error.

It can be seen from the unit root test results in Table 8 that the data series have a mixed integration, meaning that they are stationary at mix of I (0) and I (1). In this situation, the dataset does not meet the ordinary least square (OLS) assumption, thus the pooled OLS regression will not be applied. However, other panel regression techniques could be applied.

#### 4.2.3 Multicollinearity test

We test the data series for multicollinearity. If independent variables are correlated, the regression may give a biased estimation and the results will not be reliable. The correlation matrix of the variables presented in Table 5 shows that youth unemployment data series has a high correlation with exports, population growth and agriculture. However, the result of the variance inflation factor (VIF) test in Table 6 indicates there is no serious multicollinearity in the dataset as the mean VIF is 2.90 which is below the threshold of 10 [96].

**Table 5:** The correlation matrix of the variables.

Variable	(1)	(2)	(3)	(4)	(5)	(6)	(7)	(8)
(1) Youth unemploy	1.000							
(2) FDI China	0.049	1.000						
(3) FDI others	0.158	0.222	1.000					
(4) GDP growth	-0.131	-0.087	-0.116	1.000				
(5) Domestic invest	0.132	0.173	0.449	0.052	1.000			
(6) Population gr	-0.613	-0.110	-0.171	0.173	-0.035	1.000		
(7) Human capital	0.570	0.095	0.244	-0.169	-0.011	-0.752	1.000	
(8) Inflation	-0.035	-0.115	-0.093	-0.024	-0.002	0.113	-0.091	1.000
(9) Infrastructure	0.471	0.315	0.269	-0.191	0.120	-0.484	0.622	-0.178
(10) Openness	0.599	0.278	0.216	0.021	0.424	-0.383	0.230	-0.074
(11) Agriculture	-0.627	-0.047	-0.162	0.104	-0.107	0.607	-0.653	0.011
(12) Institutional qty	0.408	0.008	0.204	-0.074	0.222	-0.643	0.666	-0.139
(13) Tech gap China	-0.484	0.182	-0.040	-0.003	-0.097	0.432	-0.413	-0.057
(14) Tech gap USA	-0.541	-0.006	-0.140	0.040	-0.186	0.522	-0.583	0.030
(15) Nat. resources	-0.203	-0.105	0.168	0.102	0.178	0.366	-0.272	0.248
	(9)	(10)	(11)	(12)	(13)	(14)	(15)	
(9) Infrastructure	1.000							
(10) Openness	0.271	1.000						
(11) Agriculture	-0.450	-0.485	1.000					
(12) Governance	0.408	0.224	-0.447	1.000				
(13) Tech gap China	-0.289	-0.262	0.560	-0.450	1.000			
(14) Tech gap USA	-0.490	-0.317	0.625	-0.478	0.872	1.000		
(15) Nat. resources	-0.311	0.044	0.095	-0.379	0.139	0.218	1.000	

**Table 6:** The VIF test results for the variables.

Variable	VIF	1/VIF
Technology-Gap USA	7.53	0.133
Technology-Gap China	6.47	0.154
Human capital	4.98	0.201
Population growth	3.14	0.319
Institutional quality	2.86	0.350
Agriculture	2.69	0.371
Infrastructure	2.13	0.471
Domestic Investment	2.02	0.496
Openness	1.95	0.514
Natural resources rent	1.67	0.598
FDI others	1.53	0.652
FDI China	1.42	0.703
Inflation	1.14	0.874
GDP growth	1.10	0.913
Mean VIF	2.90	

#### 4.2.4 Heteroskedasticity and autocorrelation tests

It is equally important to test the dataset for heteroskedasticity and autocorrelation. The presence of autocorrelation means that the error terms are correlated, which can lead to biased estimation. Heteroskedasticity shows the distribution of error terms, that is whether error terms are normally distributed or not. We apply the Wooldridge serial correlation test which can identify first-order serial correlation in panel data [97]. Previous simulations show that Wooldridge test has good size and power properties in reasonably sized samples. The Wooldridge test has as a null hypothesis that there is no autocorrelation. If the p-value is significant (i.e., P < 0.5) the null hypothesis is rejected. In other words, a significant test statistic indicates the presence of serial correlation. We assess the dataset for heteroskedasticity using a process of Likelihood-ratio (LR) test as demonstrated by Wiggins and Poi (2023) [98]. The test has as a null hypothesis that the residual variance is constant (i.e., homoskedasticity) and the alternative hypothesis that residual variance is not constant (i.e., heteroskedasticity). The null hypothesis is rejected if the corresponding p-value is significant (e.g., p-value < 0.05). The Wooldridge test for autocorrelation (Table 7) rejects the null hypothesis of no first order serial correlation with a p-value of 0.0075. Similarly, the LR test strongly rejects the null hypothesis homoscedasticity, indicating the existence of heteroskedasticity with a p-value of less than 0.01.

**Table 7:** Autocorrelation and heteroskedasticity test results of the data.

Autocorrelation: Wooldridge test	Heteroscedasticity: LR test
F (1, 33) = 8.146	Chi^2^ (40) = 987.86
Prob > F = 0.0075	Prob > Chi^2^ = 0.0000

In the presence of autocorrelation, heteroscedasticity and mixed unit root stationarity of order 0 and order 1 (i.e. after 1st difference), it is recommended in literature to apply specific panel regression techniques, such as the fixed-effects (FE) and random-effects (RE) models–with some correction commands, the feasible generalized least square (FGLS) model, the panel corrected standard errors (PCSE) model, or the generalized method of moments (GMM) [45, 99, 100].

#### 4.2.5 Estimation technique

Previous empirical studies of the employment effect of FDI have used different estimation techniques. Among the studies that used panel data, the estimation techniques include static models (e.g. FE and RE models), or dynamic models (e.g. dynamic FE, RE, GMM and autoregressive distribution lag (ARDL) models. For example, Choudhry et al. (2012) employed FE model [101], Folawewo and Adeboje (2017) employed the fully modified ordinary least square (FMOL) [79], Jude and Silaghi (2016) used the system GMM approach [26], while Mkombe et al. (2021) employed FGLS technique [45].

The tests performed on our dataset reveal the presence of autocorrelation, heteroskedasticity, and a mix of I (0) and I (1) unit roots. In this situation, it is recommended in literature to apply specific panel regression techniques, such as the FE and RE models–with some correction commands, FGLS model, the PCSE model, or the GMM model [45, 99, 100].

In our main dataset which consist of 42 SSA countries over 19 years period (2003 to 2021), the number of years is less than the number of countries (i.e., T < N). Thus, FGLS and PCSE estimators could not be used to estimate our main model because both estimators require that the number of time periods be greater than the number of individuals (i.e., T > N). The GMM estimator would be suitable for our data because it is efficient for dynamic analysis where N > T and it can use from-within instruments to control for potential endogeneity. However, preliminary estimation of our data using the more efficient Roodman two-step system GMM estimator automatically dropped many observations and failed to produce all diagnostic test results, including Sargan, Hansen, AR1 and AR2 tests, because of collinearity problem and gaps in the data series. To maintain confidence in our regression, the GMM estimator could not be used for the main analysis, however, we use a Blondell and Bond (1998) system GMM estimator to check for robustness [102]. A choice had to be made between FE and RE models, although they may be weakened by endogeneity problems. The Hausman test was used to select the appropriate estimator between FE and RE. According to the Hausman test result, RE estimator was appropriate. Thus, the RE generalized least square (GLS) regression approach is used with a correction command of robust cluster id to account for the presence of autocorrelation and heteroskedasticity.

## 5. Results and discussion

Table 8 presents the regression estimates for the effect of FDI on youth unemployment in SSA. Regression-1 in the table tests Eq (6), that is the full model without interactive variables, while regressions-2 to 5 estimate different specifications of Eq (7), which controls for FDI interactions with domestic human capital and institutional quality. The equations are also separately tested for Chinese FDI and Other FDI. Their regression estimates are presented in Tables 9 and 10, respectively. The vitals of the regression are good, with robust t-statistics and acceptable r-squared.

**Table 8:** Regression results for effect of FDI on youth unemployment in SSA.

Independent Variables	(1)	(2)	(3)	(4)	(5)
FDI China	-0.282**	-0.436*	-0.335**	-0.198	-0.229
	(0.122)	(0.300)	(0.145)	(0.140)	(0.147)
FDI Other	0.00585*	0.00607*	0.00485	-0.0102	0.00324
	(0.003)	(0.004)	(0.004)	(0.017)	(0.005)
GDP growth	-0.0381	-0.0437	-0.0416	-0.0601**	-0.0626**
	(0.025)	(0.027)	(0.025)	(0.026)	(0.026)
Domestic Investment	-0.00693	-0.00493	-0.0129	-0.00565	-0.0128
	(0.029)	(0.030)	(0.030)	(0.038)	(0.038)
Population growth	-0.161	-0.157	-0.523	-0.736	-0.815
	(0.403)	(0.448)	(0.445)	(0.522)	(0.518)
Human capital	-0.00899	-0.00714	0.000542	0.0231	0.0279
	(0.031)	(0.032)	(0.032)	(0.036)	(0.034)
Inflation	0.018	0.0184	0.021	0.0251	0.025
	(0.018)	(0.019)	(0.019)	(0.021)	(0.022)
Infrastructure development	0.0314	0.0274	0.0441	0.0366	0.0426
	(0.045)	(0.049)	(0.047)	(0.049)	(0.050)
Openness	0.0296***	0.0341***	0.0393***	0.0675***	0.0687***
	(0.011)	(0.011)	(0.010)	(0.016)	(0.018)
Agriculture	-0.0783	-0.0826	-0.102*	-0.140**	-0.141**
	(0.058)	(0.060)	(0.059)	(0.066)	(0.067)
Institutional quality	-2.203*	-2.261*	-1.911	-2.441	-2.347
	(1.233)	(1.290)	(1.289)	(1.537)	(1.669)
Technology Gap China	-0.287**	-0.281**	-0.316**	-0.326**	-0.330**
	(0.122)	(0.122)	(0.127)	(0.138)	(0.142)
Technology Gap U.S.	-0.00299	-0.00942	-0.004	-0.0258	-0.026
	(0.023)	(0.024)	(0.023)	(0.024)	(0.024)
Natural resources	-0.145***	-0.151***	-0.147***	-0.167***	-0.170***
	(0.040)	(0.042)	(0.043)	(0.051)	(0.052)
FDI China × Human capital		0.00264			
		(0.004)			
FDI China × Institutions			-0.18		
			(0.132)		
Other FDI × Human capital				0.00018	
				(0.000)	
Other FDI × Institutions					0.00347
					(0.009)
Constant	18.15***	18.29***	18.41***	17.87***	17.84***
	(3.980)	(3.946)	(4.057)	(3.968)	(3.739)
R-squared	0.512	0.533	0.578	0.618	0.619
F-test	3937***	8284***	10920***	21112***	1335***
Observations	407	407	407	407	407

Note: Standard errors are in parenthesis

*** p<0.01

** p<0.05

* p<0.1.

**Table 9:** Regression results for effect of Chinese FDI on youth unemployment in SSA.

Independent Variables	(1)	(2)	(3)	(4)	(5)
FDI China	-0.261*	-0.303**	-0.235*	-0.29*	-0.300**
	(0.142)	(0.140)	(0.133)	(0.322)	(0.137)
GDP growth	-0.0508**	-0.0527**	-0.0409*	-0.0438*	-0.0413*
	(0.0240)	(0.0236)	(0.0228)	(0.0245)	(0.0230)
Domestic Investment	-0.00967	-0.00406	-0.00246	-0.0018	-0.00828
	(0.0304)	(0.0299)	(0.0276)	(0.0286)	(0.0284)
Population growth	-0.161	-0.357	-0.108	-0.16	-0.397
	(0.480)	(0.482)	(0.405)	(0.441)	(0.427)
Human capital	-0.0192	-0.00321	-0.00453	-0.000951	-0.000194
	(0.0315)	(0.0339)	(0.0306)	(0.0319)	(0.0313)
Inflation	0.0139	0.0119	0.0192	0.0195	0.0215
	(0.0179)	(0.0178)	(0.0185)	(0.0189)	(0.0184)
Infrastructure development	0.0876**	0.0512	0.0347	0.0313	0.0469
	(0.0359)	(0.040)	(0.0397)	(0.0453)	(0.0423)
Openness	0.0203*	0.0223*	0.0316***	0.0355***	0.0365***
	(0.0119)	(0.0116)	(0.0099)	(0.0104)	(0.0093)
Agriculture	-0.0311	-0.038	-0.0787	-0.0847	-0.0941*
	(0.0659)	(0.0679)	(0.0562)	(0.0578)	(0.0565)
Institutional quality	-0.835	-1.281	-2.414**	-2.411**	-2.094*
	(1.079)	(1.133)	(1.175)	(1.221)	(1.217)
Technology Gap China		-0.224**	-0.290***	-0.305***	-0.308***
		(0.0873)	(0.0777)	(0.0774)	(0.0819)
Natural resources			-0.152***	-0.155***	-0.149***
			(0.036)	(0.0379)	(0.0377)
FDI-China × Human capital				0.000985	
				(0.0043)	
FDI-China × Institutions					-0.187
					(0.122)
Constant	12.82***	15.67***	17.35***	17.42***	17.67***
	(3.798)	(4.199)	(3.951)	(3.939)	(4.068)
R-squared	0.490	0.550	0.496	0.524	0.551
F-test	286.4***	368.2***	450.4***	575.5***	516.9***
Observations	407	407	407	407	407

Note: Standard errors are in parenthesis

*** p<0.01

** p<0.05

* p<0.1.

**Table 10:** Regression results for effect of other FDI on youth unemployment in SSA.

Independent Variables	(1)	(2)	(3)	(4)	(5)
FDI Other	0.00632*	0.00633	0.00452	-0.00924	0.00424
	(0.0038)	(0.0039)	(0.0036)	(0.0112)	(0.0032)
GDP growth	-0.0397*	-0.0416	-0.0356	-0.0347	-0.0351
	-0.0241	-0.0253	-0.0247	-0.0247	-0.0247
Domestic Investment	-0.0225	-0.0226	-0.0195	-0.00846	-0.0154
	(0.0315)	(0.0318)	(0.028)	(0.0311)	(0.0315)
Population growth	-0.0901	-0.0563	0.206	0.302	0.283
	(0.504)	(0.504)	(0.424)	(0.423)	(0.426)
Human capital	-0.0181	-0.0183	-0.0232	-0.0271	-0.0239
	(0.0327)	(0.0326)	(0.0284)	(0.0287)	(0.028)
Inflation	0.0148	0.015	0.0225	0.0229	0.0226
	(0.0172)	(0.0171)	(0.018)	(0.0181)	(0.018)
Infrastructure development	0.0819**	0.0823**	0.0797**	0.0699*	0.0747**
	(0.0384)	(0.0388)	(0.0374)	(0.0392)	(0.0397)
Openness	0.0128	0.0104	0.0203	0.0228**	0.0209*
	(0.0119)	(0.0123)	(0.0113)	(0.012)	(0.0119)
Agriculture	-0.0349	-0.0343	-0.0719	-0.0712	-0.0699
	(0.0637)	(0.0657)	(0.0546)	(0.0546)	(0.0547)
Institutional quality	-0.584	-0.75	-1.872	-2.22*	-2.116*
	(1.115)	(1.149)	(1.249)	(1.288)	(1.254)
Technology Gap U.S.		-0.0131	-0.0239	-0.0248	-0.0234
		(0.0159)	(0.0176)	(0.0179)	(0.0176)
Natural resources			-0.143***	-0.138***	-0.141***
			(0.038)	(0.0397)	(0.0388)
FDI-Other × Human capital				0.000181	
				(0.0001)	
FDI-Other × Institutions					0.00377
					(0.0059)
Constant	12.98**	13.67**	14.97***	14.79***	14.73***
	(3.993)	(4.316)	(4.006)	(4.062)	(3.996)
R-squared	0.466	0.477	0.456	0.440	0.435
F-test	1286***	644***	873***	15799***	2002***
Observations	407	407	407	407	407

Note: Standard errors are in parenthesis

*** p<0.01

** p<0.05

* p<0.1.

### Effect of Chinese FDI on youth unemployment in SSA

In Table 8 regression-1 (i.e. full model without interactive variables), the Chinese FDI variable has a negative coefficient that is significant at 5% significance level (β = -0.282; p < 0.05). This suggests that Chinese FDI has a significant negative effect on youth unemployment rate in SSA. That is, on average, youth unemployment rate in SSA decreases by 0.282 percentage points for each additional unit of Chinese outward FDI stock expressed as a share of host country GDP. In other words, if the host country in SSA attracts Chinese FDI equivalent to 10% of the host country’s GDP, the host country’s youth unemployment rate could be reduced by 2.82 percentage points. The first three regressions in Table 9 (i.e. models with Chinese FDI and no interactive terms) support the above result for Chinese FDI. Thus, all four regressions of eq-(6) confirm hypothesis one (H1) in the case of Chinese FDI. Furthermore, the regression estimates of eq-(7) which has interaction variables indicate that Chinese FDI has negative and significant effect on youth unemployment in most cases. Specifically, in Table 8 regression-2 the Chinese FDI variable has negative and significant coefficient (β = -0.436; p < 0.1), suggesting that on average, a unit increase in Chinese FDI stock as a share of GDP reduces youth unemployment by 0.44%. Similarly, the coefficient of Chinese FDI variable in Table 8 regression-3 indicates that on average, one unit increase in Chinese FDI stock as a ratio of host GDP decreases youth unemployment rate by 0.34%. In Table 9, regressions 4 and 5 also indicate negative and significant effect of Chinese FDI on the dependent variable after controlling for the impact of domestic absorptive capacity. This gives further confirmation to H1 in the case of Chinese FDI. The results are consistent with the findings of other research papers [47, 49, 91, 103, 104]. The results could partly be explained by the relatively large amount of capital investment (greenfield projects) China makes in Africa (Ernst, 2021), and the fact that large percentage of Chinese investment and development financing target infrastructure development, such as transportation and electricity infrastructure, which are the areas where many SSA countries face deficits. Chinese investment in these types of infrastructure does not only help bridge infrastructure gaps, but it also supports regional and domestic connectivity [105], which can generate jobs. In addition, Chinese FDI to Africa is increasingly targeting labor-intensive activities, such as construction and manufacturing. In recent years China has participated in construction of industrial parks, housing and other facilities in several countries. Also, its investment in manufacturing activities in Africa has increased, as China seeks for efficiency due to rising labor costs at home. Although these types of investments can lead to job losses if the focus of investment is to gain efficiency, it could still promote local industrialization and create jobs at least in the short-term [106].

In the case of Other-FDI, the coefficients of Other FDI in the regression estimates of eq-(6) are positive and too small, but significant in two cases only, including Table 8 regression-1 (β = 0.00585; p < 0.1) and Table 10 regression-1 (β = 0.00632; p <0.1). These results, with the negligible coefficients, suggest that Other FDI has a minimal increasing effect on youth unemployment in SSA. Thus, based on regression estimates of eq-(6), H1 cannot be confirmed in the case of Other FDI. The regression estimates of eq-(7) also indicate that Other-FDI has mostly insignificant and positive coefficients, except in two cases where coefficients are negative, i.e. Table 9 regression-4 (β = -0.0102; p = 0.537) and Table 10 regression-4 (β = -0.00924; p = 0.409), which suggests a reducing but insignificant effect on youth unemployment after controlling for FDI’s interaction with human capital. Based on these results, H1 could not be confirmed for Other-FDI. This could be due to more FDI entry through the M&A and privatization modes which do not often lead to job increases in the short-term, and it could be due to predominance of resource-seeking investments in Africa which do not complement the job needs of host countries. Natural resource-seeking FDI in particular often create fewer or no jobs in Africa. ILO (2020) reports that for every one million US dollar of greenfield investment, extractive industry generates only 0.6 jobs compared to 2.76 jobs created in manufacturing industry [107]. The insignificant effect could also be due to market-seeking FDI failing to complement local investment. Such FDI often leads to displacement effects, such as crowding out domestic firms by taking over investment opportunities available to them, and then immediately reorganizing and restructuring acquired firms by employing more capital and technology than labor for production. The unexpected finding regarding Other FDI is consistent with the findings of Mkombe et al. (2022) in southern Africa region [45], Inekwe (2013) in Nigeria’s service industries [108], and some of the findings of Massoud (2008) in Egypt [109].

### Lagged FDI

The effect of FDI on employment may manifest with a time lag [20]. Therefore, the lagged values of FDI are also estimated. We estimate 2-year, 3-year, 4-year, and 5-year lags of FDI. We report results for the 3-year lag (Table 11). Regression results of other lags are available upon request. The results indicate that lagged Chinese FDI has coefficients that are positive and statistically insignificant in most cases, except in regression-3. In the case of Other-FDI, the coefficients are small but positive and statistically significant in most cases. Thus, we note that lagged FDI does not have a reducing effect on youth unemployment in SSA. The unexpected result for Chinese FDI could be explained by several factors. Firstly, there is limited transfer of technology from Chinese firms to host countries due to weak linkages with domestic firms. One of the channels through which FDI promotes long-term output and employment growth in host countries is through the transfer of technology and demand effect. This depends on the extent of the technology gap and the linkages between Chinese and domestic enterprises. If the technology gap is not too wide and linkage occurs between Chinese and domestic firms, there will be technology transfer which can result in output and employment growth through positive demand effects, movement of employees from Chinese firms to domestic firms, and shared supplier strengthening. But without linkages, none of these beneficial effects would be realized. As we can see from all the regression results, technology gap with China appears to have significant negative effect on youth unemployment, yet lagged Chinese FDI is insignificant, meaning that absence of linkages between Chinese and domestic enterprises explains the result. This is consistent with the findings of Cooper (2019) which surveyed the empirical literature on the effect of Chinese investment in Africa. She reported that “there is a consensus that Chinese investment has weak backward linkages (related to the modalities of Chinese investment) and can act as “enclaves,” unintegrated with the host country’s economy. This can inhibit positive local economic development outcomes [109]. Secondly, the jobs created by Chinese investment in the construction industries may be unsustainable. Although Chinese investment in infrastructure development creates jobs directly, such jobs might be lost in the future. Construction jobs involve many roles such as manual labor, architecture, designing, civil engineering and so on. Many Chinese firms in the construction industry tend to hire the architect, designer and engineers from China while local workers are hired in manual labor roles. The manual labor roles often lack long-term career perspective and training as they are easily downsized depending on availability of construction project contracts. In addition, several studies report that some Chinese investors, especially private-owned Chinese firms tend to “ignore local laws, hire workers without contracts or dismiss employees arbitrarily, causing frequent labor disputes” [110]. This can negatively affect the employment outcomes of Chinese FDI in the long-term. Our results in this regard are consistent with other scholars’ outcomes [18, 53].

**Table 11:** Regression results for effect of lagged FDI on youth unemployment in SSA.

Independent Variables	(1)	(2)	(3)	(4)	(5)
FDI China(t-3)	0.258	0.434**	0.269	0.259	0.266
	(0.204)	(0.218)	(0.204)	(0.207)	(0.209)
FDI Other(t-3)	0.00404*	0.00361	0.00367	0.00336*	0.00409*
	(0.002)	(0.002)	(0.002)	(0.002)	(0.002)
GDP growth	-0.0271	-0.0428	-0.029	-0.0268	-0.0268
	(0.027)	(0.027)	(0.027)	(0.027)	(0.027)
Domestic Investment	-0.0251	-0.0312	-0.028	-0.0253	-0.0228
	(0.025)	(0.026)	(0.027)	(0.025)	(0.028)
Population growth	-0.162	-0.424	-0.242	-0.182	-0.133
	(0.345)	(0.370)	(0.345)	(0.350)	(0.335)
Human capital	-0.0306	-0.0203	-0.0283	-0.0314	-0.0302
	(0.022)	(0.024)	(0.023)	(0.022)	(0.022)
Inflation	0.0178	0.0193	0.0189	0.0181	0.018
	(0.020)	(0.020)	(0.020)	(0.020)	(0.020)
Infrastructure development	0.0553	0.0639*	0.0588	0.0551	0.0522
	(0.037)	(0.039)	(0.040)	(0.038)	(0.040)
Openness	0.0116	0.0212**	0.014	0.0121	0.0122
	(0.009)	(0.009)	(0.009)	(0.009)	(0.010)
Agriculture	-0.0692	-0.0952	-0.0807	-0.0718	-0.0675
	(0.064)	(0.066)	(0.067)	(0.064)	(0.064)
Institutional quality	-2.572*	-2.351*	-2.44*	-2.534*	-2.738*
	(1.337)	(1.306)	(1.327)	(1.353)	(1.417)
Technology Gap China	-0.152	-0.169	-0.155	-0.152	-0.156
	(0.109)	(0.117)	(0.105)	(0.109)	(0.109)
Technology Gap USA	-0.0104	-0.0224	-0.0128	-0.0113	-0.00964
	(0.027)	(0.027)	(0.025)	(0.027)	(0.027)
Natural resources	-0.166***	-0.167***	-0.168***	-0.166***	-0.165***
	(0.044)	(0.045)	(0.045)	(0.046)	(0.045)
FDI China × Human capital		-0.0036**			
		(0.001)			
FDI China × Institutions			-0.0421		
			(0.107)		
Other FDI × Human capital				0.0000167	
				(0.000)	
Other FDI × Institutions					0.00215
					(0.005)
Constant	18.30***	19.25***	18.58***	18.47***	18.11***
	(4.084)	(4.044)	(4.126)	(4.088)	(4.019)
R-squared	0.347	0.484	0.389	0.345	0.348
F-test	1121***	1337***	1932***	5478***	3742***
Observations	340	340	340	340	340

Note: Standard errors are in parenthesis

*** p<0.01

** p<0.05

* p<0.1.

### The interactive terms

The regressions in Tables 8 and 9 indicate that current Chinese FDI interaction with human capital have coefficients that are positive and statistically insignificant, and much smaller than the individual FDI. This result is unexpected as it is inconsistent with our prior theoretical expectation and suggests the lack of complementarity between human capital and Chinese FDI in reducing youth unemployment in SSA in the immediate term. The implication of this result is that local human capital interaction with Chinese FDI has not immediately enhanced the reducing effect of Chinese FDI on youth unemployment. Perhaps if host countries have appropriate levels of human capital stock, the reducing effect of current FDI on youth unemployment would be much stronger. In other words, the human capital conditions of host countries have limited the potential youth employment benefits of Chinese FDI. Therefore, this result fails to confirm H2 which states that the effect of Chinese FDI on youth unemployment in SSA depends on the host country’s stock of human capital. However, the interactive term of Chinese FDI and institutional quality has coefficients that are negative but statistically insignificant, as seen in regression-3 of Table 8 (β = 0.18; p = 0.173). Although this result has the expected sign, the effect is negligible. More importantly, the coefficient is smaller than individual institutional quality and Chinese FDI. This result suggests the existence of weak complementarity between institutional quality and Chinese FDI in reducing youth unemployment. In other words, the effect of individual Chinese FDI on youth unemployment is not strongly enhanced by its interaction with local institutional quality. Thus, this finding fails to confirm H3 which states that the effect of Chinese FDI on youth unemployment in SSA depends on the host country’s institutional quality. Overall, interactive terms of current Chinese FDI have insignificant moderating effect in the relationship between Chinese FDI and youth unemployment. With regards to Other FDI, the interactive terms with human capital and institutional quality have coefficients that are positive and statistically insignificant. More importantly, the coefficients are much smaller than individual institutional quality, human capital and Other FDI. These results conflict with our prior theoretical expectation and points to a lack of complementarity between human capital, institutional quality and other FDI in reducing youth unemployment in SSA. Similar to the conclusion in the case of Chinese FDI, the regression results here fail to confirm H2 and H3 in the case of Other FDI.

Overall, our regression estimates suggest that individual current Chinese FDI has a reducing effect on youth unemployment and this effect does not depend much on interactions with host country human capital and institutional quality. However, overtime, the effect of individual Chinese FDI on youth unemployment becomes statistically insignificant. The reasons are already discussed in previous chapter. This long-term effect also does not depend on human capital and institutional quality. Other FDI has overall insignificant effect on youth unemployment and there is no moderating effect from its interaction with host country human capital and institutional quality. Thus, with regards to H4, the regression results suggest that the effect of Chinese FDI on youth unemployment in SSA differ from that of Other FDI only through the short-term direct effect. Overtime, the direct and indirect effects of Chinese FDI and Other FDI on youth unemployment in SSA are not significantly different.

Our results also reveal the impact of GDP on youth unemployment. As can be seen in all the regressions, the estimated coefficients of the GDP variable are negative in all cases and statistically significant in many cases. This corroborates Mkombe et al. (2022) and Folawewo and Adeboje (2017) [45, 79]. In Africa, employment growth usually lags behind GDP growth [106]. This is due to several reasons. Firstly, most African countries depend heavily on low value-added activities (such as mining, oil and gas, and commodity exports) for their economic growth. These activities are not labor-intensive, and they pay low wages to the small number of local employees utilized. Thus, it is difficult to generate positive demand effects and employment growth from the growth obtained from these extractive activities. Secondly, the manufacturing sector, which should generate more jobs, makes only small contribution to the GDP in most African countries. Indeed, data shows Africa’s manufacturing GDP as a share of total GDP is much lower compared to other developing regions. Thus, less job is created as a result of total GDP growth. Thirdly, part of economic expansion in Africa is due to population growth and not necessarily as a result of structural change [106]. This fast population growth adds more pressure on the already rapidly growing young labor force. Thus, the number of jobs created from GDP growth will only have negligible impact on the huge labor supply. Fourthly and most importantly, significant share of Africa’s labor-force is still absorbed in mostly subsistence agriculture, thus even a relatively large contribution of agricultural industry to total GDP cannot generate sufficient jobs. The results also show that domestic investment has a reducing effect on youth unemployment, but rather insignificantly, mainly because investment rate in SSA is typical insufficient.

With regards to Technology gap China, the results indicate the coefficients is negative and statistically significant in all cases, except the case of lagged FDI. This suggests that technology gap with China strongly reduces youth unemployment in SSA. This could be due to the closeness or suitability of Chinese technology to the technology level of host countries, which makes it easier for the local workers to be recruited immediately by Chinese firms. Or it could be that Chinese firms are immediately training local employees to upgrade their skills to Chinese technology level. It could also be that Chinese firms, as mentioned earlier, absorbed local workers in positions that do not require advanced skills. However, as the technology gap narrows, the effect on youth unemployment becomes insignificant. This is indicated by the statistically insignificant coefficients of technology gap China in Table 11. Natural resources also have coefficient that is negative and statistically significant in all cases. This suggests that natural resources endowment has significant reducing effect on youth unemployment in SSA. This is not surprising, considering that many young people engage in crude activities, which they tend to identify as their occupation. With regards to openness, the coefficient is positive and statistically significant in all cases. This suggest that openness of the economy to international trade is strongly associated with increased youth unemployment. This could be due to inability of SSA countries to harness the potential benefits of economy openness for its development as a result of weak competitiveness, such as insufficient policies, inadequate infrastructure, inefficient institutions, failure to exploit access to global value chains, and substitution effect on domestic firms due to hurting of local industry.

The results indicate that the coefficients of inflation are positive but statistically insignificant in all cases. This suggests that inflation has a weak positive effect on youth unemployment, meaning that youth unemployment increases with inflation. This nullifies the Phillips-Curve hypothesis which suggests the existence of inverse relationship between inflation and unemployment. The weak positive effect can be partly explained by the high inflation experienced in most SSA countries during the study period. As wages adjust to inflation, businesses are discouraged from expanding and creating new job positions, leading to slower employment growth. This suggests the need to pursue low to moderate inflation policy. With regards to agriculture, we note that the coefficient is negative in all cases but statistically significant in some cases only. This suggests that agricultural productivity has a weak reducing effect on youth unemployment in SSA. This is partly due to the sector’s low productivity and the informal nature of agricultural labor in many SSA countries, some of which are not captured in statistics. Indeed, it shows that agriculture can be a major source of employment. This suggests the need for more investment in agricultural infrastructure, and the training and education of both farm and non-farm workers, so as to increase agricultural output, and hence employment. Population growth has negative coefficient that is statistically insignificant in most cases, suggesting weak reducing effect on youth unemployment, contrary to prior theoretical expectation for developing countries. One reason for this could be that the growth in population mainly affect young age group in which majority of the members are below the working age and not competing for employment. Indeed, SSA is known as the world’s youngest region [1]. Another reason could be that the growth in population creates new consumers which increases market demand, and thus investment and job opportunities.

With regards to human capital, the coefficient is negative in most cases, but statistically insignificant. This suggests that human capital level reduces youth unemployment in SSA but not significantly. This could be due to shortage of skilled labor and poor healthcare, suggesting the need for SSA countries to prioritize investment in education, healthcare and training of workers.

Institutional quality has significant reducing effect on youth unemployment in most of the cases, confirming the importance of efficient institutions in realizing output and employment growth. We also estimate Eq (6) with six individual good governance indicators (Table 12). The results indicate that all six variables have coefficients that are not significant statistically. However, the coefficients of control of corruption, government effectiveness, regulatory quality, and voice and accountability are negative, suggesting a weak reducing effect on youth unemployment, while political stability and rule of law have positive coefficients, suggesting an increasing effect on youth unemployment in SSA. The results also confirm earlier results regarding Chinese FDI and other FDI.

**Table 12:** Regression results for the impact of Chinese OFDI on youth unemployment in SSA, 2003–2021, with individual governance indicators.

*Independent variables*	Full Model with all FDI		Model with Chinese FDI		Model with other FDI	
FDI China	-0.312	(0.137) **	-0.243	(0.147) *		
FDI other	0.007	(0.004) *			0.006	(0.004)
GDP growth	-0.048	(0.024) **	-0.062	(0.025) **	-0.030	(0.023)
Domestic invest	0.002	(0.029)	0.000	(0.029)	-0.004	(0.026)
Population growth	-0.571	(0.460)	-0.846	(0.546)	-0.197	(0.434)
Human capital	0.016	(0.031)	0.039	(0.033)	0.007	(0.030)
Inflation	0.024	(0.020)	0.029	(0.022)	0.024	(0.019)
Infrastructure	0.039	(0.043)	0.053	(0.043)	0.039	(0.044)
Openness	0.043	(0.011) ***	0.066	(0.013) ***	0.026	(0.011) **
Agriculture	-0.083	(0.055)	-0.114	(0.058) **	-0.064	(0.050)
Tech Gap China	-0.349	(0.132) ***	-0.359	(0.146) **	-0.292	(0.128) **
Tech Gap USA	-0.021	(0.023)	-0.029	(0.023)	-0.007	(0.025)
Natural resources	-0.140	(0.039) ***	-0.162	(0.040) ***	-0.140	(0.033) ***
Corruption control	-1.770	(1.361)	-1.213	(1.430)	-1.439	(1.386)
Govt effectiveness	-1.333	(1.361)	-1.527	(1.353)	-1.396	(1.408)
Political stability	0.548	(0.572)	0.518	(0.602)	0.375	(0.586)
Rule of law	0.943	(1.742)	0.705	(1.766)	1.055	(1.695)
Regulatory quality	-0.165	(1.393)	-0.211	(1.336)	-0.446	(1.490)
Voice & accountability	-1.515	(1.097)	-1.617	(1.206)	-1.258	(1.097)
Constant	17.362	(3.848)	16.095	(3.838)	15.934	(4.303)
R-squared (overall)	0.569		0.6186		0.494	
Wald x2	26293***		2617***		514.14***	
Observations	407		407		407	

Note: Standard errors are in parenthesis

*** p<0.01

** p<0.05

* p<0.1.

We also conduct separate regressions to account for significant adverse economic events that occurred during the study period. Usually, significant events like global scale economic recessions can have an impact on cross-border capital flows, investment, production and labor demand (employment). When testing the impact of investment on employment, it is important to control for this kind of event, so as to produce a more reliable result. Two main global scale economic events have occurred during the period of this study, the global financial crises (GFC) of the late 2000s, which began in December 2007 and ended in June 2009, and the COVID-19 economic recession of 2020 that lasted several months in most countries. To control the effect that the GFC may have on the relationship between Chinese outward FDI and employment in SSA, we drop observations for year 2008 and 2009, and for Covid-19 recession, we drop all observations for year 2020. Table 13 presents the regression results. In both the full model and model with Chinese FDI, the coefficient of Chinese FDI is negative and statistically significant. In the full model (β = 0.30; p<0.05), the result indicates that in general, a unit increase in Chinese FDI stock as a ratio of GDP is associated with 0.30 percentage point decrease in youth unemployment in SSA. This confirms our earlier results in this study, and implies that the economic recessions did not affect Chinese FDI’s impact in SSA. In the case of Other FDI, the coefficient is positive and statistically significant in both cases. This suggests a strong increasing effect on youth unemployment in SSA. The implication is that economic recessions adversely affect the flow and impact of Other FDI in SSA.

**Table 13:** Regression results for the impact of Chinese OFDI on youth unemployment in SSA, 2003–2021, controlled for GFC and COVID-19 recession.

*Independent variables*	Full Model with all FDI		Model with Chinese FDI		Model with other FDI	
FDI China	-0.300	(0.123) **	-0.249	(0.129) *		
FDI other	0.008	(0.003) ***			0.007	(0.004) *
GDP growth	-0.047	(0.031)	-0.055	(0.032) *	-0.046	(0.030)
Domestic invest	-0.007	(0.028)	-0.001	(0.028)	-0.014	(0.028)
Population growth	-0.153	(0.445)	-0.173	(0.453)	-0.082	(0.522)
Human capital	-0.010	(0.029)	0.000	(0.030)	0.001	(0.032)
Inflation	0.007	(0.013)	0.009	(0.013)	0.011	(0.013)
Infrastructure	0.016	(0.048)	0.026	(0.047)	0.020	(0.050)
Openness	0.045	(0.011) ***	0.050	(0.011) ***	0.046	(0.011) ***
Agriculture	-0.098	(0.051) *	-0.108	(0.052) **	-0.116	(0.047) **
Institutional quality	-2.084	(1.121) *	-2.353	(1.107) **	-2.045	(1.160) *
Tech Gap China	-0.340	(0.124) ***	-0.341	(0.127) ***	-0.327	(0.130) **
Tech Gap USA	0.010	(0.022)	0.007	(0.023)	0.011	(0.024)
Natural resources	-0.164	(0.040) ***	-0.174	(0.040) ***	-0.178	(0.040) ***
Constant	18.401	(3.922)	17.457	(3.807)	17.206	(4.201)
R-squared (overall)	0.554		0.565		0.555	
Wald x2	493.15***		442.75***		362.56***	
Observations	352		352		352	

Note: Standard errors are in parenthesis

*** p<0.01

** p<0.05

* p<0.1.

To further check the analysis for robustness, we estimate Eq (6) and different specifications of Eq (7) using the Arellano–Bover/Blundell–Bond linear dynamic panel-data estimation, a system GMM estimator proposed by Blundell and Bond (1998) [102]. The estimator uses moment conditions based on the level equations together with the orthogonality conditions that exist between lagged values of the dependent variable and the disturbances. The estimator is also suitable for our dataset of N > T. In addition, the vce (robust) option is applied to address biased standard errors. Table 14 presents the regression estimates. As can be seen, in most cases, the results confirm earlier results in this study. Chinese FDI has a reducing effect on youth unemployment through the direct effects and indirectly through its interaction with local institutions. In contrast, Other FDI has significant positive effect on youth unemployment in most cases both directly and indirectly. This confirms the robustness of results of the main estimations.

**Table 14:** Regression results for effect of FDI on youth unemployment in SSA using Blundell and Bond (1998) system GMM estimator.

Independent Variables	(1)	(2)	(3)	(4)	(5)
Youth Unemployment(t-1)	0.798***	0.791***	0.790***	0.799***	0.803***
	(0.092)	(0.094)	(0.095)	(0.091)	(0.090)
FDI China	-0.19*	-0.0639	-0.244**	-0.166	-0.14
	(0.138)	(0.201)	(0.118)	(0.140)	(0.149)
FDI Other	0.0119***	0.0119**	0.0121**	-0.00373	0.0082
	(0.004)	(0.005)	(0.005)	(0.016)	(0.006)
GDP growth	-0.0166	-0.0167	-0.0164	-0.0174	-0.0171
	(0.023)	(0.023)	(0.023)	(0.024)	(0.023)
Domestic Investment	-0.0148	-0.0164	-0.0179	-0.0125	-0.0152
	(0.025)	(0.026)	(0.025)	(0.026)	(0.023)
Population growth	-0.817	-0.849	-0.874	-0.836	-0.796
	(0.587)	(0.569)	(0.568)	(0.594)	(0.598)
Human capital	0.0781*	0.0834**	0.0807**	0.0710**	0.0764**
	(0.032)	(0.034)	(0.032)	(0.034)	(0.033)
Inflation	-0.0189	-0.019	-0.0196	-0.0182	-0.0174
	(0.017)	(0.017)	(0.017)	(0.017)	(0.017)
Infrastructure development	0.01	0.0119	0.0122	0.00881	0.0076
	(0.012)	(0.013)	(0.012)	(0.011)	(0.011)
Openness	-0.00497	-0.0052	-0.00559	-0.00353	-0.00204
	(0.006)	(0.006)	(0.006)	(0.006)	(0.006)
Agriculture	-0.0856	-0.0834	-0.0813	-0.0862	-0.0873
	(0.065)	(0.067)	(0.065)	(0.065)	(0.065)
Institutions	-2.634***	-2.687***	-2.339**	-2.670***	-3.026***
	(0.941)	(0.934)	(0.937)	(0.954)	(1.114)
Technology Gap China	-0.202**	-0.223**	-0.229**	-0.174*	-0.162*
	(0.095)	(0.097)	(0.093)	(0.095)	(0.097)
Technology Gap U.S.	0.0302	0.0303	0.0315	0.0264	0.0261
	(0.020)	(0.020)	(0.019)	(0.022)	(0.021)
Natural resources	-0.0216	-0.0212	-0.0183	-0.0208	-0.0221
	(0.033)	(0.033)	(0.033)	(0.033)	(0.034)
FDI China × Human capital		0.00233			
		(0.004)			
FDI China × Institutions			-0.165**		
			(0.082)		
Other FDI × Human capital				0.000187	
				(0.000)	
Other FDI × Institutions					0.0128
					(0.010)
Constant	1.821	1.796	2.097	2.231	1.665
	(2.695)	(2.772)	(2.689)	(2.715)	(2.638)
Wald X2	3370***	4557***	4410***	4489***	123.17***
Observations	386	386	386	386	386
Countries	39	39	39	39	36
Instruments	49	50	50	50	33
AR2 p-value	0.792	0.828	0.831	0.807	0.530

Note: Standard errors are in parenthesis

*** p<0.01

** p<0.05

* p<0.1.

## 6. Conclusion

This study was conducted to estimate the effect of Chinese outward FDI on youth unemployment in SSA using robust RE GLS panel estimator, based on data obtained from WDI, UNCTAD, and CARI for 42 SSA countries over the period 2003–2021. The study reveals as follows. Firstly, current Chinese FDI has a significant reducing effect on youth unemployment, while Other FDI has mostly insignificant effect on youth unemployment. This finding is explained by the fact that large share of Chinese capital investment targets infrastructure development which is lacking in many SSA countries, thus Chinese FDI can better complement domestic investment efforts in stimulating immediate employment growth compared to Other FDI. The finding is also explained by the fact that Chinese FDI has higher concentration in labor-intensive activities, such as construction and manufacturing, compared to Other FDI which has higher concentration in extractive activities and capital-intensive manufacturing and services. Moreover, China’s capital flows to SSA tends to use the greenfield entry mode compared to Other FDI which has more M&As and privatization entry modes. Secondly, lagged Chinese FDI and lagged Other FDI (three or more years ago) do not have significant effect on current youth unemployment in SSA. This is explained by lack of demand effect due to absence of linkages between FDI firms and domestic firms, and due to lack of stable and rewarding employment in the case of Chinese FDI. Thus, there is minimal employment growth through the channels of positive demand effects, movement of employees and shared supplier strengthening. However, sometimes the failure of linkages is due to inability of host countries to absorb resources provided by more advanced foreign firms. Thirdly, the host country human capital and institutional quality play a weak modulating role in the relationship between Chinese FDI and youth unemployment in SSA. Finally, the employment effect of Chinese FDI and Other FDI in SSA differ only in the immediate direct effect, their indirect and long-term effects are generally the same.

To realize positive employment outcomes from inward FDI, SSA countries should pursue a labor absorbing FDI policy that attracts more greenfield FDI from China and other FDI sources. The greenfield projects should be channeled to agriculture, manufacturing, infrastructure construction, and other sectors that have the capacity to generate more employment opportunities. More investment should be guided to agriculture, especially in areas of agricultural infrastructure and agribusiness education [111] to increase productivity because increased productivity in Agric sector is strongly associated with employment growth in an African economy [112, 113], as large number of youths in the region are directly or indirectly linked to agriculture. Policy efforts should also focus on facilitating effective interaction between foreign and domestic enterprises to promote indirect job creation as the result of vertical linkages and positive horizontal learning and competition effects. In addition, governments of SSA should further strengthen their institutions, invest in human capital, and increase the quantity and quality of infrastructure so as to attract and support private domestic and foreign investments, better absorb the potential benefits from FDI, stimulate economic activities, and create sustained employment growth. The public infrastructure investment should be guided to areas where the region is facing deficits, such as transportation, water and sanitation, schools and hospitals, roads, and energy. Policymakers should also implement pro-business policies and regulations in specific sectors (such as agriculture, tourism, and energy), reduce natural resource exploitation, and diversify the economies, to create employment opportunities that can utilize the growing young labor-force.

It is important to point out the possible limitations of this study. While efforts have been made to correct for the presence of autocorrelation and heteroscedasticity in the data, the panel analysis using the RE GLS technique may be limited by endogeneity issues. Although the Blundell and Bond (1998) system GMM estimator has been used for robustness check, we recommend further investigation with more advanced and efficient estimators as more data is accumulated [101].
